# Making the medical mask: surgery, bacteriology, and the control of infection (1870s–1920s)

**DOI:** 10.1017/mdh.2022.5

**Published:** 2022-04

**Authors:** Thomas Schlich, Bruno J. Strasser

**Affiliations:** 1 Department of Social Studies of Medicine, McGill University, 3547 Peel Street, Montreal, QC H3A 1X1, Canada; 2 University of Geneva, Geneva, Switzerland

**Keywords:** Mask, Surgery, Bacteriology, Asepsis, Hygiene, Infection

## Abstract

This article examines the introduction of the medical mask in the late nineteenth century at the intersection of surgery, bacteriology and infection control. During this important episode in the longer history of the medical mask, respiratory protection became a tool of targeted germ control. In 1897, the surgeon Johannes Mikulicz at the University of Breslau (now Wroclaw, Poland), drawing on the bacteriological experiments of his colleague Carl Flügge, used a piece of gauze in front of his nose and mouth as a barrier against microorganisms moving from him to his patients. This article explores the social, cultural and medical contexts of this particular use of the mask, in connection with germ theory and surgeons’ struggle with wound infection. It explores the alignment of the new aseptic surgery with the emerging field of bacteriology in a local milieu that favoured interdisciplinary cooperation. The account also follows the uptake of the mask outside of surgery for other anti-infectious purposes and shows how the new type of anti-infectious mask spread simultaneously in operating rooms as well as in hospitals and sanatoria, and eventually in epidemic contexts.

In today’s age of biomedicine, it is still a simple nineteenth-century technology, the medical mask that constitutes one of the first lines of defence against infectious disease. This article investigates an important episode in the longer history of the medical mask to examine how it emerged, in the late nineteenth century, at the intersection of surgery and bacteriology as a tool of targeted germ control. In 1897, the surgeon Johannes Mikulicz at the University of Breslau (now Wrocław, Poland), drawing on the bacteriological experiments of his colleague Carl Flügge, used a piece of gauze in front of his nose and mouth as a barrier to keep microorganisms from moving from the surgeon to the patient. The assumption was that a single microorganism emitted by the surgeon’s breath could kill a patient. Mikulicz did not invent the medical mask, but he gave a new meaning to an old sanitary practice. This meaning has persisted, as surgical masks and medical masks in other contexts are still used today for protecting others from infection by healthy carriers, and medical masks are evaluated as to their ability to warrant maximum sterility.^1^

The practice of covering one’s face and nose as a method for the prevention of disease has a long history. The materiality of this practice – mostly a simple piece of cloth tied around the head – has shown remarkable stability.^2^ The mask is an example of an old technology whose usefulness persists, and which is worth historical attention despite the usual priority of innovation in historiography.^3^ Behind their material continuity, masking practices have undergone important changes in meaning. This article explores the context in which one of these changes occurred. We first consider some of the developments of masks before 1897, especially the beginnings of the connection between germ theory and masking. We then describe the surgeons’ long-standing struggle with wound infection, and how, in this struggle, the perfect control of microorganisms became the dominating strategy. For analysing the place of masks in this strategy, it is useful to examine in detail how surgery and the emerging field of bacteriology crossed paths in the local context where the surgical mask first emerged. This shows how Mikulicz’s quest for perfect control led him to take recourse to the kind of bacteriologically informed hygiene, which his colleague Carl Flügge was developing at the time. For him, bacteriology control was a plausible and attractive strategy for dealing with the risk of wound infection. For Flügge, in turn, surgery offered another area of application for his specific approach to hygiene. As we show, this interaction happened within a local cultural milieu that favoured this kind of exchange. Finally, we follow the immediate uptake of the mask outside of surgery for other anti-infectious purposes, first locally, through Flügge’s laboratory, but very quickly also at a national and international level. The new type of anti-infectious mask thus spread simultaneously in operating rooms as well as in hospitals and sanatoria before becoming a piece of personal protection in times of epidemics.

## Masks and germs

In the early nineteenth century, masks were rarely used for protection against epidemic diseases. But starting in the 1860s, some of the proponents of the existing germ theories began to recommend employing masks against contagion by these microscopic organisms.^4^ The Irish physicist John Tyndall, an early convert to Pasteur’s germ theory and perhaps the most widely read advocate of germ theory in the Anglophone world, illustrates this trend. In 1870, he devised a cotton wool ‘respirator’ to intercept ‘floating matter on its way to the lungs’.^5^ He started developing respirators ‘side by side’ with the spread of germ theory, as he phrased it, and subsequently included germs in the list of noxious influences that needed to be kept from entering the body through the respiratory tract or the digestive system.^6^

Tyndall had been converted to the germ theory of disease by the work of Louis Pasteur, but also by his own optical experiments showing that seemingly clean air contained numerous barely visible particles, which he identified as ‘dust’ and ‘germs’. For Tyndall, the germ theory offered a new framework to understand sanitary practices, such as wearing a mask: ‘we have revealed to us the true philosophy of a practice followed by medical men, more from instinct than from actual knowledge. In a contagious atmosphere, the physician places a handkerchief in his mouth and inhales through it. In doing so he unconsciously holds back the dirt and germs of the air’. Tyndall’s cotton wool respirator was intended to protect patients as well as physicians from ‘the germs by which contagious disease is said to be propagated’.^7^

Tyndall explicitly rejected linking his device ‘indissolubly with the germ theory’ because, as he wrote, he did not want to prejudice the opponents of germ theory. This was essential since his respirator was also an important means of keeping other matters out of the lung, such as the ‘stony grit’ deposited in the lungs of stone cutters, and stopping the irritation and fever that affected seedsmen in Lancashire. The respirator could also be used as protection from cold air or be employed for moistening the respiratory tract to prevent irritation of the throat and coughs.^8^ To protect firefighters from smoke and sewer workers from miasma, Tyndall made a special respirator, for which he used cotton wool as a filter, glycerine for catching atmospheric germs and charcoal for neutralising smoke.^9^ Thus, Tyndall envisioned this sanitary practice as a way of preventing the inhalation of a broad spectrum of noxious matters in the air, sometimes including germs.

In the late 1870s, new masks focused more narrowly on preventing the inhalation of germs. In 1879, for example, the French physician Henri Henrot recommended a respirator for preventing infection. He based his considerations on the Pasteurian version of germ theory according to which air-borne bacteria would get into the body through the lungs and cause fermentation of the patient’s blood. Therefore, bacteria had to be kept out of the respiratory passages. Henrot suggested a type of cone to be worn in front of the nose and mouth, in which the inhaled air passed through a layer of cotton wool. It could be particularly useful for physicians treating patients with croup, he claimed. Two of Henrot’s interns who had been afflicted by fever and foetid diarrhoea after conducting autopsies in the hot summer of 1874 were able to avoid these incidents by using his respirator, he claimed.^10^

Around the same time, in Germany, the botanist Carl Nägeli speculated about the usefulness of masks for filtering out air-borne bacteria. Nägeli was known for his microscopic studies of different kinds of cells including the role of what he called *fission fungi* – later replaced by the term ‘bacteria’ – in the causation of infectious disease. In his 1877 textbook about this topic, he discussed the danger of infection by these fungi carried by the dust particles in the air. He claimed that the only effective means of protection consisted in intercepting the dust particles with a filtering device in front of the nose and mouth. He suggested using a wet sponge with fine pores, multiple layers of wet cloth, or a respirator which could be kept moist by using glycerine or, more pragmatically, a simple pad of cotton wool pushed against the mouth by a means of a rag placed tightly around the mouth and nose. Such filters could be worn on special occasions, when visiting the sick, for example, when just going ‘for an errand in a city that was struck by an epidemic’, or by nurses who were in contact with patients suffering from diseases such as diphtheria and ‘acute exanthema’.^11^

Louis Pasteur himself also embraced the idea of an anti-germ mask. In 1879 at the French Academy of Medicine, he stated that he would not fear to study the ongoing plague in Egypt if, among other precautions, he could cover his mouth and nose with a cotton mask.^12^ However, his suggestion was met with vivid criticism. The physician Jules Rochard believing ‘to be the spokesperson for the great majority of physicians’, claimed ‘that none will agree to saddle themselves with a cotton mask to approach their patients’. For Rochard, who was a broad anticontagionist, in times of epidemics ‘such precautions would be impracticable or illusory’.^13^

In general, rejection or acceptance of masks was not determined by the views about germ theory, but by other factors. Most of the opposition to masking came from people who believed in exclusive contact transmission (or transmission through water), rather than aerial contagion. In 1879, the French commentator Ferdinand Delauney mocked Henrot’s mask as a useless and ridiculous muzzle (a reference to the metal mesh of many respirators). Germ theory, which he apparently despised, ‘in all logic will lead us to live like divers in an underwater suit (“*scaphandre*”).’^14^ By contrast, the physician Emile Decaisne believed in germs, but he also found masks useless because germs, ‘smart as they are’ would simply find another path to enter the body. In 1884, in the popular weekly *L’Univers Illustré*, he mocked Pasteur’s mask by comparing it to the beaked plague mask. He hoped that ‘fear and stupidity would not lead his tough and glorious profession to become a public laughingstock’.^15^ Decaisne’s position was originally elaborated in reference to cholera, which was now increasingly considered to result from water contamination. Often physicians’ attitudes towards masking practices in times of epidemics seemed to have resulted more from their familiarity with specific diseases than from their general views about germ theory. In fact, masks could be rationalised as a technology for holding back miasma, as well as germs present in the air. This broad theoretical grounding of masking practices narrowed down drastically at the end of the century. The context of this more specific perspective was surgery.

## Antisepsis and asepsis

By the mid-nineteenth century, operative surgery was in deep crisis. Practitioners observed an outbreak of wound disease of pandemic proportions, as historians describe it, in hospitals across Europe and the United States^16^: Days after a successfully treated injury or a felicitous operation, the patient’s wounds would show signs of inflammation and start secreting pus. Sometimes, the affected body part would die off and had to be amputated. Often, general symptoms, such as fever or prostration appeared, and many patients eventually died from these septic complications. This seemed to happen more frequently than ever before. Doctors were unsettled by this development and reacted in different ways.

Some practitioners followed a multi-pronged approach to address different possible causes of wound disease.^17^ Thus, they improved their surgical techniques to optimise the healing process; they made sure that their patients were in good health and that their surgical work environment was clean, well-organised and tidy. Others followed an even broader ‘sanitarian’ strategy. They thought of wound infection in a public health mode as a place-dependent disease. The place that caused wound disease was the hospital, where, as it seemed, most wound complications happened. For the sanitarians, the way to fight wound disease was to replace the large urban hospitals with smaller units that were well-aired, easy to control and amenable to frequent rebuilding. This strategy would, however, deprive surgeons of an important place for operative performance and training.

In 1867, Joseph Lister suggested a more targeted approach that would avoid closing down the surgical wards and operating theatres of hospitals. Lister postulated that wound disease was caused by the germs of the same microscopically small living entities that had been identified by Pasteur in 1863. These would multiply in the patients’ wounds and lead to putrefaction. Lister and his adherents directed all their attention to these germs. They claimed that if the germs were killed by using disinfectants, such as carbolic acid, wound disease could be prevented and even cured with certainty. Wounds had to be protected from the air-borne germs by gauze dressings, soaked in carbolic acid, that would both intercept and eliminate the germs out of the surrounding air. This targeted strategy was called ‘antisepsis’. Adopted by many practitioners during the second half of the nineteenth century, it eventually became a standard method, though both the theory and the practice underwent considerable changes over time. Antisepsis represented a narrow, technical approach to solving the problem of wound disease, which had now been identified as being caused by microorganisms.

With antisepsis, hospital reform was no longer necessary. The general environment became almost irrelevant, as did the general condition of the patient. It was enough to use carbolic acid to control germs wherever they were. Lister himself used a carbolic acid spray to disinfect the air in the operating venues. Yet, he never mentioned masks, gloves, gowns, or any other elements of the later surgical garb. On the contrary, he kept operating in his old frock coat and did not attach great importance to cleanliness. Interestingly, others made the connection to the mask. For Tyndall, Lister applied the same principle of ‘the filtering power of cotton wool’ to the ‘treatment of wounds’, as he himself did with his mask.^18^ Lister himself saw the antiseptic dressing as a kind of filter. In 1871, he described how dry cotton wool alone effectively prevented ‘by its filtering property, the access of any putrefactive agent’, but since in its application to wounds the wool got wet and permeable to microscopic organisms Lister impregnated it with carbolic acid.^19^ Henrot too referred to antiseptic dressing as the model for his type of mask.^20^

The approach to wound infection changed again when, in the 1880s, surgeons in the German-speaking countries went one step further in the direction of targeted germ control. They recommended a new strategy, which they called ‘asepsis’. Asepsis took Robert Koch’s germ theory as its basis. According to Koch, putrefaction was just one symptom of the infection of the wound or, in the worst case, of the patient’s entire body, which occurred through the open wound. Like other infections, it was caused by specific microorganisms that produced specific diseases by invading the body. More important than Koch’s theory, however, were his new techniques for identifying and tracing bacteria. They provided a new generation of aseptic surgeons with the tools for their strategy of tracing down and avoiding any infectious germs in the first place instead of killing them with disinfectants.^21^ This seemed to work well at first. However, in the late 1890s, many aseptic surgeons became concerned because the new strategy turned out to be extremely precarious.^22^ They had noticed that their efforts could not always guarantee that the operation wound stayed free of germs, as Mikulicz noted in 1898. The tiniest breach in the aseptic condition could result in wound infection. Even worse, the surgeon added, the transition from antisepsis to asepsis had apparently not improved the surgical outcomes. This observation put the whole project of asepsis into question, and it prompted many investigators to assiduously search for weak points in the current methods of antiseptic wound treatment.^23^ This was the context in which Mikulicz began the explorations that lead to the surgical mask.

## Surgery in Breslau

Mikulicz had trained in Vienna with one of the leading surgeons of the time, Theodor Billroth, and subsequently acted as chief of surgery in Kracow and Königsberg (today Kaliningrad, Russia). Since 1890 he was the head of the surgery department of the University of Breslau. Breslau was a burgeoning industrial city and a major centre of medical science and intellectual life. Undergoing a process of rapid industrial growth, the city benefited from its proximity to the heavy industry of Upper Silesia. One of the city’s most important industrial products was railway carriages and engines. In 1913, the local railway manufacturing company celebrated the completion of its one-hundredth locomotive. Breslau’s university was the fifth largest in Germany, home to 2 000 students and 189 academic staff. Its scientists and doctors made discoveries that shaped modern medicine. One of the early pioneers of bacteriology, Ferdinand Cohn, was the director of the Institute of Plant Physiology where the then-unknown Robert Koch worked for a year. The director of the Dermatology Clinic was another celebrated bacteriologist, Albert Neisser, who worked on leprosy and syphilis, but had his name permanently linked with the bacteria that causes gonorrhoea, *Neisseria gonorrhoeae*, which he discovered in 1879.^24^ As it was typical for the German-language universities of the time, the university consisted of relatively autonomous and well-funded academic units with a strong commitment to research. The heads of the clinics and institutes enjoyed almost complete independence from the universities and the state. This applied also to Mikulicz, who was described as being very much king in his own realm.^25^ At Breslau he had a brand-new aseptic operation theatre at his disposal, equipped with an instrument cooker, and a steriliser for dressing material, which enabled him to make the move from antisepsis to asepsis. He also established new chemical, bacteriological and pathological laboratories in his clinic,^26^ and introduced a division of specialised expertise among his assistant surgeons with special training in pathological anatomy, internal medicine, bacteriology, chemistry or physics.^27^ The ‘Breslau surgical school’ soon became one of the most productive and prestigious places of surgical training in Germany.^28^

Mikulicz belonged to a generation of academic surgeons who had adopted Koch’s bacteriology in the 1880s.^29^ In several publications in 1897 and 1898, he discussed how the switch from antisepsis to asepsis had increased the stakes. Antisepsis had been relatively forgiving. It followed what Mikulicz called the ‘exterminator’ or ‘pest control principle’ by killing germs indiscriminately. By contrast, asepsis followed a much more targeted strategy of avoiding any contamination from the patient’s environment. The downside was that, as he put it, ‘the smallest mistake in wound treatment would come back to haunt the surgeon in a much more serious way than in the past’. It was all or nothing now. Mikulicz felt he either had to give up the aseptic principle altogether and return to the old antiseptic practices, or he had to work on the refinement of asepsis ‘to its uttermost consequences’.^30^ He went for the latter, which meant the control of all possible sources of contamination. All objects, for example, used in an operation – swabs, compresses, and so forth – became possible sources of contamination.^31^

Control was the dominating theme in Mikulicz’s work. At his clinic, he organised work with military strictness. When he entered the hospital, the janitor had the task to pull a bell three times to announce the chief’s arrival. From that point on, nobody was allowed to leave the hospital until he himself had left.^32^ The assistant physicians had to assemble immediately and present Mikulicz a report about the condition of the critically ill patients. Mikulicz generally scheduled his first operation for 9 o’clock in the morning, and his staff was to be punctual. The tone of communication in the clinic was clipped and to the point, strictly confined to medical matters. Mikulicz did not like to see the doctors in his clinic greet each other by handshake, nor did he allow them to address each other with the usual professional courtesy of ‘Herr Kollege’.^33^ He was short-tempered and choleric, someone who did not mince words, and did not accept excuses or justifications in cases of mistakes or negligence.^34^ He kept an eagle eye on any gesture and any fleck of dust. As a matter of routine, more operations than could possibly be conducted were scheduled, with the purpose of avoiding idleness, even if it was only for a few minutes.^35^ Obsessed with efficiency and time management, he always seemed to be overbooked by his multiple activities in the clinic and outside.^36^ The work environment at Mikulicz’s clinic was thus shaped by the principle of control and by his overbearing autocratic personality.

Mikulicz was fascinated by modernity For him, surgery was a modern technology, like the railway, mining or the metal industry.^37^ Mikulicz invented surgical instruments and technical devices, such as an operating table, an anaesthetic inhaler, and the first usable endoscope.^38^ Mikulicz deeply believed that technology could fix most problems. The laboratory-inspired control strategy of asepsis itself was a technological fix that relegated other potential factors of wound healing to the background. Aseptic surgeons focused on those things that they knew could control. Uncontrollable factors, such as the healing power of nature, were of no interest to them. When Mikulicz spoke about the natural protective mechanisms of the patient’s tissues, he only did it to stress that it was ‘very limited; the less we expect from it, the surer we can be of success, the more powerful operative surgery is going to be’.^39^

## Hygiene in Breslau

Mikulicz thought that to achieve the goal of perfect sterility, surgical expertise alone was no longer sufficient. He found it indispensable to co-operate with an expert on hygiene and infectious diseases. Locally, Carl Flügge was such an expert. Flügge was another powerful head of a university unit, the Institute of Hygiene. Within the setting of the German research universities of the time, such a collaboration was not unusual. It was facilitated by social and cultural proximity within the milieu. Mikulicz and Flügge belonged to the same caste of academic civil servants, with close connections to the military, not only in spirit. Flügge had participated in the French–Prussian war in 1870/71. Mikulicz served his year of military service as a doctor in 1873, though not in the armed forces. Subsequently, he became a physician in reserve (Reservearzt) of the Austrian Militia (Landwehr), but had to resign from the Austrian Army, when he moved to Germany later.^40^ In Breslau, he was appointed Generalarzt à la suite, which was a military title given to those who were allotted to the army or a particular unit for honour’s sake and entitled him to wear a regimental uniform, but otherwise had no official position.^41^ Mikulicz and Flügge moved in the same academic-intellectual milieu of the local bourgeoisie. The focus of Breslau’s polite society was Neisser’s villa. At his house, he entertained not only doctors and scientists but also, among others, the writer and future Nobel laureate Gerhart Hauptmann, as well as the composers Gustav Mahler and Richard Strauss. Mikulicz also took part in his parties.^42^ An anecdote about Flügge illustrates how at Breslau the fences between the disciplines and institutions were low and the distances short. One night in the early 1890s, a colleague of Flügge’s received a visit from the institute servant of the neighbouring Institute of Hygiene, which Flügge was heading, with a note from the director. A barrel of oysters had been stranded there, the note said, and he needed to come over immediately to help save it. The rescue operation, which was doused with a considerable amount of champagne, turned into a very enjoyable evening.^43^ We do not know if Flügge and Mikulicz socialised in this way, maybe not, but the episode shows the culture of sociability within the academic milieu. In any case, Mikulicz was no stranger to interdisciplinarity and often collaborated with non-surgical colleagues. He had, for example, established an important interdisciplinary journal with a leading German internist with the title ‘Notes from the Borderlands of Medicine and Surgery’.^44^

Flügge was one of the first scientists to adopt Koch’s methods of visualising and culturing bacteria in order to determine their species and applied Koch’s techniques to examine the problems of hygiene that he investigated. He had obtained his postdoctoral habilitation degree in the discipline of hygiene at the University of Berlin in 1878, and subsequently served as the founding director of the first Prussian institute for hygiene in Göttingen, before he became a professor at Breslau in 1887. Flügge was a leading public health expert and one of the founders of the laboratory-based discipline of bacteriology. Together with Koch, he established the *Zeitschrift für Hygiene* in 1885 and wrote several authoritative textbooks in the new field.^45^ In 1897, when he first reported about his ongoing collaboration with Mikulicz, Flügge’s popular textbook on hygiene and public health, was in its fourth edition.^46^

**Figure 1:**
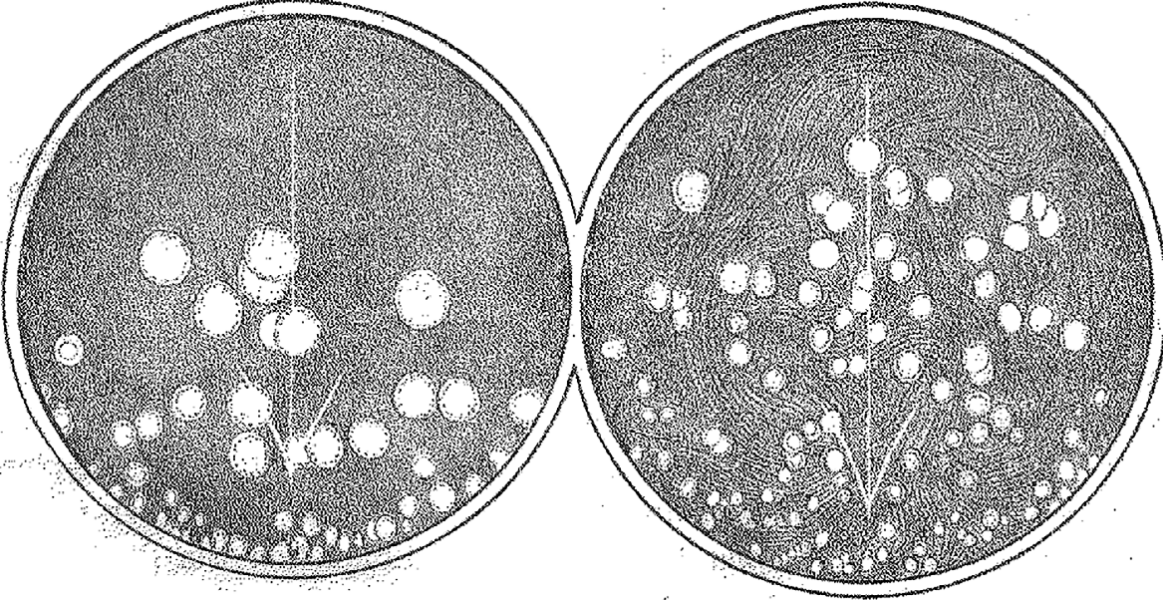
Bacillus prodigiosus colonies used by Flügge for investigating droplet infection. C. Flügge, ‘Ueber Luftinfection’, *Zeitschrift für Hygiene und Infektionskrankheiten*, 25, 1 (1897), 179–224, see p. 211.

One of Flügge’s research themes concerned the transmission of infectious agents from person to person through the air under different conditions. He discussed various possible germ carriers, including dust. In analogy to dry dust particles, he also looked at very small drops of water, droplets (‘*Tröpfchen*’) and tried to determine their role in infection under varying circumstances, for example, in closed rooms and in the open air. Considering the infectious role of particles in the air, such as dust, was not new, as it was precisely Tyndall’s discovery of their presence in 1870 that had become for many a powerful demonstration of the germ theory of disease. However, some doubted that the particles revealed by Tyndall’s optical methods were in fact infectious. Flügge, with his bacteriological methods, aimed to settle the question of whether air-borne particles, especially droplets, contained infectious germs. He was able to grow bacterial colonies from such particles and thus present convincing evidence for their role as carriers of living germs (Figure 1).^47^

For his experiments, he used the typical set of Kochian bacteriological tools, including solid culture media, dyes, microscopes and experimental animals. Flügge and his laboratory team established an experimental model using *Bacillus prodigiosus* as indicator species for tracking the transmission of germs. *Prodigiosus* was harmless and its colonies were bright red and thus easy to see.^48^ He followed the traditional research agenda of the field of hygiene and examined the presence and spread of germs in different kinds of spaces, producing voluminous quantitative data, including many kinds of measurements, for example, about the speed of droplets expelled by a cough and the number of germs in relation to the dimensions of rooms. In his own experiments, he exposed contaminated objects, such as clothes, to well-defined air currents that he created and used bacterial cultures to measure the number of germs that were transferred by the air. He varied the conditions using different objects, fluids or surfaces, and experimented with dust and droplets produced from different fluids sprayed into the air stream. Through these experiments, Flügge’s work showed that droplets constituted a source of contamination, even under conditions that simulated everyday behaviours like coughing, sneezing and even speaking. Based on the *Bacillus prodigiosus* model, he speculated that many diseases could be spread in this way, some of them more easily, such as smallpox, measles and tuberculosis.^49^

In these experimental explorations, Flügge did not yet include the operating room as one of the places where droplet infection might play a role. However, it was while conducting this line of investigation that he approached Mikulicz about exploring the practical significance of droplet infection in aseptic surgery, using his laboratory methods and models.^50^ Flügge even joined the surgeon in his operating room to evaluate the aseptic measures to be introduced there.^51^ This relationship is an example of the alignment of surgery and laboratory science of the 1880s and 1890s, when in many places, surgeons and bacteriologists worked side by side, taking problems from surgical practice and translating them into laboratory models. They solved these problems in the artificial world of the laboratory, where phenomena are easier to control and do not expose patients to risks, before extending the conditions of the laboratory to the operating room, that is, by adjusting surgical practices to their laboratory findings.^52^

## The surgical mask

**Figure 2:**
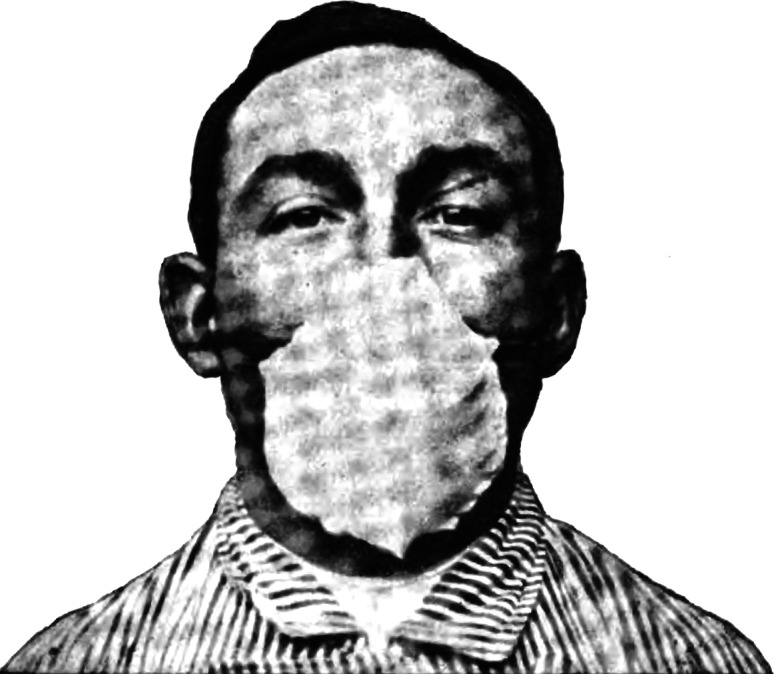
Hübener’s mask. 357. Wilhelm Hübener, ‘Ueber die Möglichkeit der Wundinfection vom Munde aus und ihre Verhütung durch Operationsmasken’, *Zeitschrift für Hygiene und Infektionskrankheiten*, 28 (1898), 348–72, see p. 357.

Through their experiments, Flügge and Mikulicz identified the droplets that were emitted by the operator’s mouth and nose as a potential gap in sterility of the operating room. One possibility to fix this gap would be to avoid speaking, a precaution already adopted by some surgeons. As Mikulicz noted: ‘In Breslau we have the habit of almost not speaking at all during surgery, the necessary communication is done through hand signals’. However, oral communication was sometimes unavoidable, for example, when a surgeon requested a particular instrument from an assistant: ‘in the end a word needs to be said from time to time’, Mikulicz acknowledged.^53^ To prevent the spread of droplets, he came up with the idea of wearing a face mask covering nose, mouth and beard.^54^ The new ‘mouth bandage’, as he called it, consisted of a simple layer of gauze and could also be used to contain the operator’s beard (the beard was back in fashion in the second half of the nineteenth century; both Mikulicz and Flügge had one). According to Mikulicz, he and his colleagues quickly got used to the new piece of equipment and, as he claimed, in gendered language and probably with some exaggeration, ‘Now we are breathing through it as easily as a lady who wears a veil in the street’ (Figure 2).^55^

Mikulicz’s surgical assistant, Wilhelm Hübener, was commissioned to conduct further laboratory work on masks or, as he phrased it, taking his chief’s cue on the language: ‘a veil made of the finest gauze to be tied in front of mouth and nose’. Mikulicz instructed him to examine the bacteriological impact of the use of masks.^56^ Following Koch’s bacteriological approach, Hübener, like Flügge before him, aimed at identifying the specific germs that were causing infections. Unlike Pasteur and Nägeli, for whom bacterial species were less important, Flügge had identified *staphylococci* and *streptococci* in the flora of the oral cavity as a source of ‘specifically surgical germs’. Likewise, Hübener proceeded to identify the specific germs that caused infection after surgery in his colleagues’ mouths, among them notably *staphylococcus* and *streptococcus.* He then tested different filters in numerous experiments using the *Bacillus prodigiosus* model. In one of the experiments, he asked his colleagues to vigorously rinse their mouth with a suspension of the bacilli. This might sound simple, but it was in fact asking a lot from his colleagues. The fluid had the nauseating taste of a brine of decomposed herring and ingesting it caused considerable revulsion. The next step was less taxing, though maybe a bit boring, as the human subjects had to count loudly up to 550, with their head slightly tilted towards a plate with agar culture medium placed below at a defined distance. He then counted the number of *prodigiosus* colonies that would grow on the plates. He varied the volume and speed of the speaking and tried out different filters. As a result, he found that a double layer of gauze was needed to keep back the bacteria. In an interesting cross-over between different aspects of surgery, he presented a mask that he had developed on the basis of Friedrich von Esmarch’s chloroform mask for anaesthesia with a wire frame and two earpieces, which had been in practical use for 6 months prior. Anaesthesia suggested itself as a possible model for building masks because it was one of the rare medical practices that routinely involved such a device. Mikulicz himself had been testing different models of anaesthetic masks.^57^ Of course, in anaesthesia, the goal was to efficiently introduce a substance into the patient’s body, not to prevent something from entering or exiting the organism.

**Figure 3:**
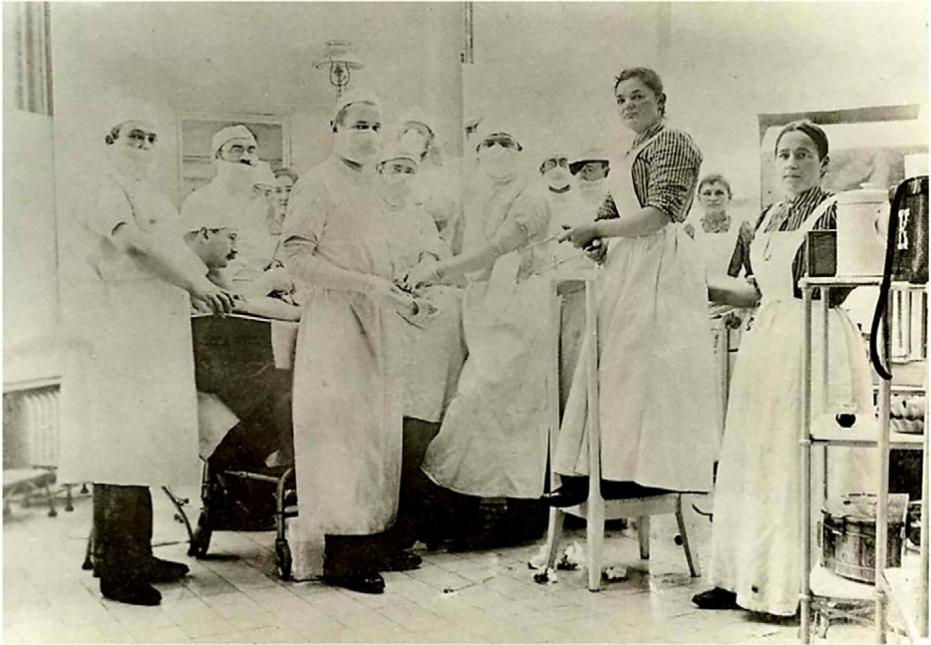
Mask as part of a system: Johannes von Mikulicz in an operating room at the University of Breslau in 1899 wearing a gauze mask and elbow-length cotton gloves. S. Hiki and Y. Hiki, ‘Professor von Mikulicz-Radecki, Breslau: 100 years Since His Death’, *Langenbeck’s Archives of Surgery* (2005), 183; reproduced with kind permission of Sumiko Hiki, Tokyo, Japan.

At first, the idea of wearing face masks was foreign to surgeons. When one of the leading American surgeons, William Williams Keen read an article by Mikulicz on surgical gloves in 1898, he asked Mikulicz to send him a pair: writing and reading was not enough to spread this new technology; the material objects themselves needed to be passed on too. Mikulicz enclosed his newly invented surgical mask along with the gloves. The American surgeon described this unfamiliar piece of surgical equipment as ‘a piece of gauze tied by two strings to the cap and sweeping across the face so as to cover the nose and mouth and beard’ (Figure 3).^58^

The face mask was part of a whole set of new measures to improve surgical sterility. They formed a comprehensive system of preventing surgical infection, which included sterile gowns and the surgical gloves that he had introduced in 1896. Many of the elements that would become part of the standard surgical paraphernalia of the twentieth century were thus added during the laboratory-driven search for weak points in the aseptic all-or-nothing system of preventing wound infection in the 1890s.^59^ This does not mean that everybody was convinced of the usefulness of such a narrow and technology-oriented strategy. The Austrian surgeon Alexander Fraenkel, for example, scorned the aseptic outfit in 1898 as ‘a whole surgical costume with a bonnet, mouth mask and veil, devised under the slogan of total wound sterility’. The exclusive emphasis on germs embodied in these precautions he judged unscientific. Local wound conditions, the role of the host organism (it could be the patient or an experimental animal) and its tissues should not be neglected, he argued. Bruises, foreign bodies, excessive blood accumulation would all lead to a ‘disposition for infection’. Very frequently, he pointed out, wounds healed perfectly despite the presence of enormous amounts of germs. It was therefore wrong to focus only on contamination by germs and neglect the processes of wound healing and more generally the bodily processes leading to the patients’ recovery.^60^ This was a common criticism of aseptic surgery as being too narrow an approach to infections because it ignored other relevant factors. Views such as Fraenkel’s represented, in a way, the opposite of Mikulicz’s all-or-nothing approach. Their existence shows that Mikulicz’s strategy was just one particular way of solving the problem and that alternative approaches circulated at the time.^61^

**Figure 4:**
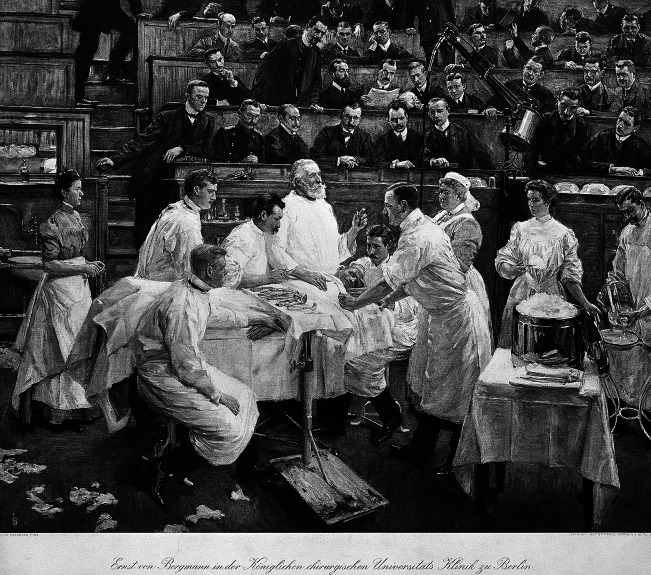
The local culture of asepsis at Ernst von Bergmann’s hospital in Berlin. Painting, 1906 by Franz Skarbina (1849–1910). Wellcome Collection.

At that time, it was not rare for surgeons to discuss the significance of bacteriologically defined sterility for their work^62^ and weighed the gains in sterility of the various precautionary measures against the loss of convenience and manual control.^63^ In practice, the use of specific protection gear varied from place to place, from surgeon to surgeon, dependent on the case and the surgical procedure. Surgeons combined different elements of the aseptic apparatus as they saw fit, creating local cultures of asepsis. In Königsberg (Kaliningrad), for example, the head surgeon adopted Mikulicz’s cotton gloves, though not the face mask.^64^ Even the surgical university clinic in the Ziegelstrasse in Berlin headed by Ernst von Bergmann was an example of another variation in local culture, despite its reputation as being the birthplace of standard aseptic technology. In a celebratory 1906 painting, Bergmann is depicted in an operation with many of the paraphernalia of aseptic surgery, such as an autoclave and surgical gowns. However, surgical masks were not among them, nor were gloves or bonnets.^65^ Some surgeons adopted specific elements of the aseptic systems, others did not (Figure 4).

**Figure 5:**
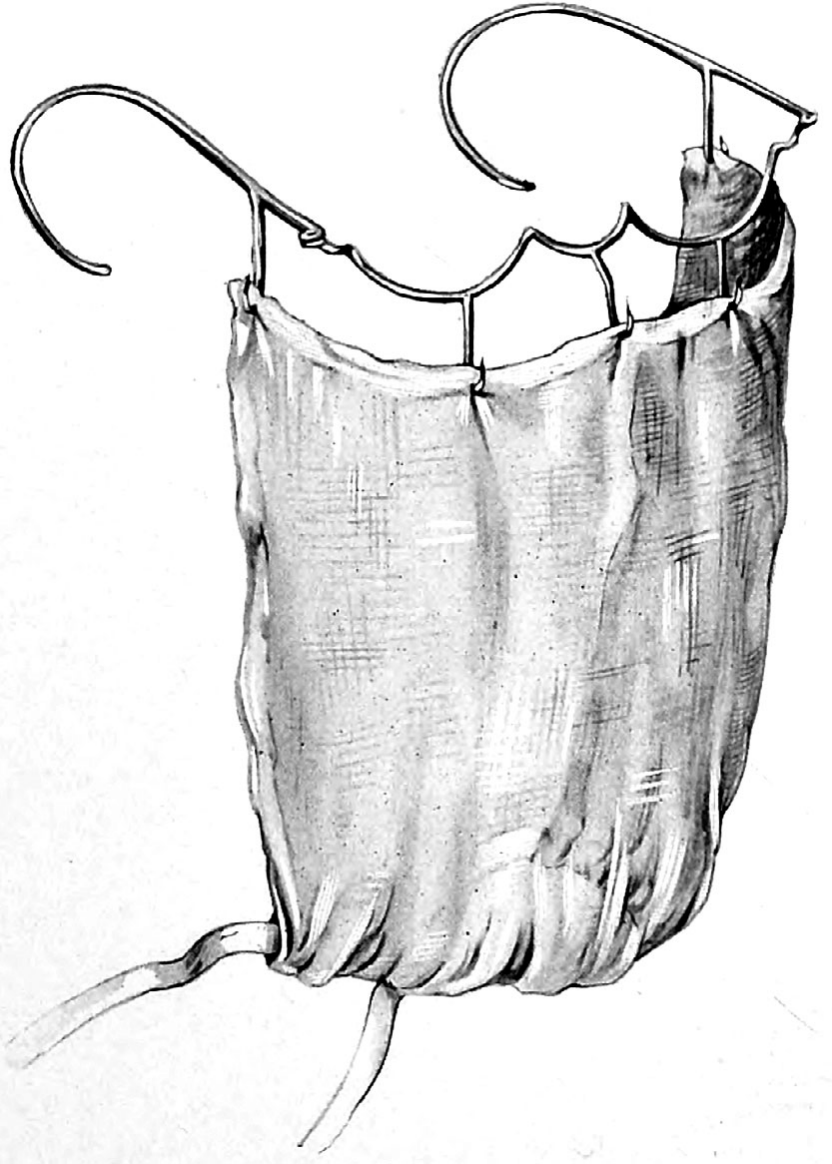
Moynihan’s mask. Berkeley Moynihan, *Abdominal Operations* (Philadelphia: Saunders, 1906), see p. 26.

However, in the longer run, masks were increasingly used in the operating rooms in different countries, often justified by reference to bacteriological science. In his 1904 textbook on abdominal operations, the influential British surgeon Berkeley Moynihan described a face mask that was fixed to a spectacle frame. Experimental work, he explained, had amply shown that ‘particles of saliva are ejected during ordinary conversation to a considerable distance’, and even worse, ‘the saliva contains organisms in profusion’. He added that, according to one eminent bacteriologist, in this respect saliva was ‘worse than the worst London sewage’ (Figure 5).^66^

After 1900, young doctors could increasingly find descriptions of masks in their surgical textbooks.^67^ At first, masks were mentioned as a kind of gadget and referred to as a ‘mouth bandage’, a ‘respirator’, or a ‘veil’, something that some surgeons used occasionally. Later editions of the same textbooks recommended masks for special circumstances, for example, in case the surgeon has a serious cold, or as an American surgeon put it with ironic distance, if the operator was ‘bearded like the pard [a legendary animal with a mane], suffers from a hacking catarrh, and sprays into the boundless universe while he talks, or if he has the habit of addressing the wound in a confidential manner’.^68^ Masks started to be offered in the catalogues of manufacturers of medical products. In 1906, the Down Brothers’ *Catalogue of Surgical Instruments* did not contain any protective masks. In 1910, it offered five different models of ‘face mask for use while operating’.^69^

Judging from photographs of operating teams in action, masks were consistently worn by most surgeons from the mid-1930s onwards, and by the 1970s the rate of surgeons wearing them reached a 100 per cent.^70^ After WWII, the question was no longer whether a mask should be worn, but what specific model was the most appropriate.

## Masks and bacteriology

Almost immediately after the surgical mask was introduced in the operating room, researchers explored the possible uses of the mask in preventing the transmission of diseases in other contexts. Grounded in theories and practices of bacteriology, such explorations focused on other infectious diseases, such as tuberculosis, scarlet fever or influenza, and other places, such as the hospital wards or the sanatoria. Researchers first examined tuberculosis, a chronic, widespread and incurable disease, which was considered one of the most important medical problems of the time. Since Koch had shown tuberculosis to be caused by a specific bacillus in 1882, the mechanisms of disease transmission could be investigated by bacteriological methods. Furthermore, since it was a bacterial disease of the respiratory tract, it was not too far-fetched to expect it to be spread through droplets.^71^

**Figure 6:**
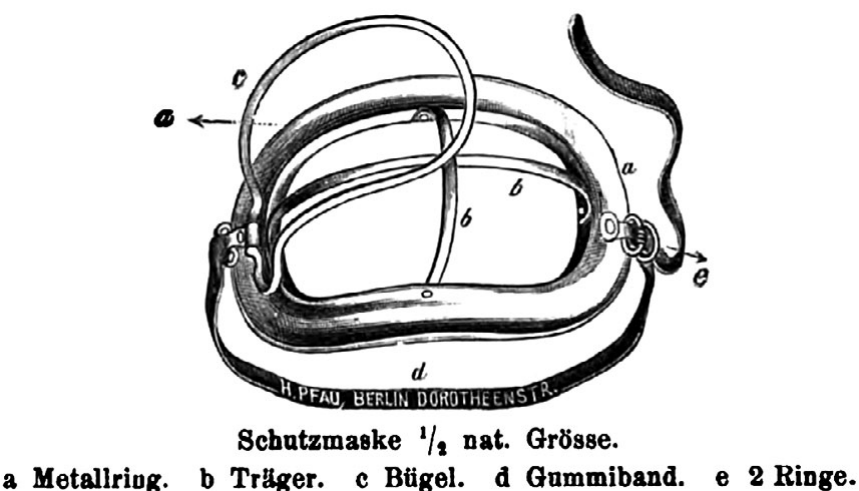
Fränkel’s mask. Bernhard Fränkel, ‘Zur Prophylaxe der Tuberculose’, *Berliner Klinische Wochenschrift*, 2 (1899), 21–6, see p. 24.

Flügge was at that time carrying out a broader research agenda on the airborne transmission of germs. In this context, he directed a study on tuberculosis patients which showed that this disease could also be spread through droplets.^72^ In one experiment, three patients were instructed to cough for 5 hours into the opening of a box containing a guinea pig. In his report, Flügge noted that, at the time of his writing, one of the animals had already died of ‘inhalative tuberculosis’.^73^ In parallel experiments, Hübener found out that patients wearing a face mask emitted considerably less bacteria from their mouth and nose.^74^ In 1897, he thus recommended that physicians *and* patients use masks as a method of prevention against the spread of various infectious diseases (Figure 6).^75^

In 1899, the physician Bernhard Fränkel at the Charité in Berlin, referred to Flügge’s bacteriological experiments and methods when discussing protection against tuberculosis.^76^ Knowledge about the spread of tubercle bacilli by droplets, he noted, had changed his views on necessary protective measures. The hotel room, the railway carriage, the clothing worn and all other items that had been in physical contact with the consumptive patient had become less suspicious, as Fränkel emphasised as a conclusion from Flügge’s results. It was now the droplets that were catapulted in the air by the cough of the sick person that required all the attention. This called for new measures, among them the use of face masks. Fränkel presented a special mask he had invented, inspired by a mask used for administering chloroform in anaesthesia (like Hübener before him), and which was produced by the instrument maker H. Pfau in Berlin.^77^ Fränkel’s mask was relatively rigid, with a metal grid as its structural basis. It was intended to be used by tuberculosis patients to prevent them from spreading germs in their environment. Fränkel moistened the material with pine needle and mint oil to give the patient the impression that the mask had an additional therapeutic effect, which, as he noted, does actually occur with such oils anyway. This impression would increase the uptake of the mask, because people were more willing to do things for their own good rather than for others, he mused.^78^

In 1900, Hermann Koeniger, a physician in Halle, was also inspired by Flügge’s experiments on the droplet infection of tuberculosis. In his own experiments, Koeniger distinguished the spread of bacteria following the pronunciation of different letters. The sounds ‘P’, ‘pr’ and ‘spr’ were very productive of droplets, he noted. This was why he had grown fond of having his subjects declaim the first verses of the Odyssey (in its original Greek: ándra moi énne*p*e, moûsa, *pol*ýtro*p*on, hòs mála *poll*à *pl*ánchthē, e*p*eì Troíēs hieròn *ptol*íethron é*p*ersen) for his tests. He also found that the homely Saxonian dialect was less ‘dangerous’ with regard to droplet infection than the sharp, dashing accent of Northern Germany. From his review of the literature, Koeniger concluded that the droplet mechanism was important for many different diseases, such as tuberculosis, pneumococcal pneumonia, diphtheria, leprosy, influenza, plague, whooping cough, as well as diseases that were caused by *streptococci* and *staphylococci.* He thus agreed with Fränkel’s suggestion that patients should wear gauze filters covering nose and mouth to prevent the spread of infections in the hospital. Interestingly, Koeniger made the parallel to the laboratory explicit by pointing to a variety of practices in the laboratory that were usually followed to avoid droplet infection in this controlled environment. Bacterial cultures, he noted, should never be ‘spoken on’ and should always be held at an angle, so that droplets from the scientists’ mouth would not drop in. However, he did not mention the use of masks in laboratories.^79^

Flügge’s *Bacillus prodigiosus* model was widely used in Germany by the researchers who worked on droplets, and even by critics in order to provide for comparability. One critical voice, who worked in Georg Gaffky’s bacteriological laboratory in Giessen, argued against masks because pathogenic organisms would dry in the mask and re-infect the sick person who was wearing it.^80^ But even these critics argued within the confines of Kochian bacteriology and its methods.

Flügge’s doctrine of droplet infection was also taken up internationally.^81^ In the first decade of the twentieth century, his approach was adopted in the United States and became the starting point for an American strand of research into droplet infection and face masks. Some of this research was about the use of masks in surgery, but much of it was about infectious diseases in other contexts. The mask, the droplet infection theory, and the *Bacillus prodigiosus* model travelled together as a package. In Chicago, several researchers worked on face masks using Flügge’s concept of droplet infection as well as his laboratory model. Alice Hamilton, known today as a pioneer of occupation health, was one of them. At the time she was a medical researcher at the Memorial Institute for Infectious Diseases and a resident at Hull House in Chicago. She had been trained in bacteriology in Munich in 1895–96 and reported, in 1905, about the experiments she conducted on droplet infection of *streptococci* in both surgical sepsis and in scarlet fever, thus bringing together surgical and non-surgical infections.^82^ From her results, Hamilton concluded that patients who were sick with scarlet fever should wear a gauze mask to protect others from being infected and that nurses and surgeons should wear masks to protect patients in the operating room.^83^

By 1910, face masks were being used by nurses, physicians, and patients in American tuberculosis clinics (always with reference to Flügge’s work as their scientific rationale). As an anonymous author in the *American Journal of Nursing* pointed out, ‘A visitor to a busy tuberculosis clinic is usually impressed by the nurses’ seeming indifference’ to their own health, because neither the patients nor the nurses themselves wore masks. Given Flügge’s results, the author recommended that nurses follow the practice of the Milwaukee, Wis., County Tuberculosis Hospital, where nurses did already ‘wear oblong mouth masks made of several thicknesses of gauze … The mask is moistened with weak disinfectant and tied over the mouth while the nurses are giving bedside care or dusting the wards’. This the author thought, was a perfect measure for institutions where all patients were infectious. In dispensaries, which were visited by tuberculous and non-tuberculous patients, however, the patients should be the ones who wear the mask, the author suggested.^84^

Similarly, the Durand Hospital in Chicago provided masks to nurses and doctors since 1916, and later to patients, in order to protect them against cross-infection.^85^ As a physician noted, ‘the mask not only protects the healthy person from infection and from becoming a carrier, but also prevents a carrier from spreading the infection to others’.^86^ Two decades after Mikulicz’s first use of the mask in the operating room, this device had become a dual-use technology intended to protect the wearer or people nearby, sometimes both. Some authors, however, worried about the excessive faith placed in masks. In 1918 Archibald Hoyne, the chief of staff at the Cook County Contagious Disease Hospital in Chicago and professor of medicine at Rush Medical College, warned against using masks as a panacea at the expense of other, more important precautions, especially hand washing.^87^ Yet, that same year, American military surgeons noted that ‘the use of face masks’ against the spread of ‘respiratory infection has now become general’, for protection in both directions. And the efficiency of different types of masks was still tested, still using Flügge’s original *Bacillus prodigiosus* model.^88^

After moving from the operating room to the hospital wards, anti-germ masks begun to be adopted in epidemic situations too. This further spread is beyond the scope of this article, but surgical masks were used during the so-called Manchurian plague in 1910 in China,^89^ sometimes in connection with the *Prodigiosus* model as a method to demonstrate their usefulness.^90^ Masks were thus still linked to the same laboratory practices, even in the context of an epidemic in a colonial setting, far away from European or North American laboratories. In fact, in the same epidemic context, researchers recommended masks on the basis of their laboratory examinations which they had conducted using a standardised pre-packaged set of bacteriological equipment ordered from a German manufacturer.^91^ So, in this instance, the mask spread literally in tandem with the scientific method proving its rationale. By the time the 1918 influenza pandemic broke out in Europe and in the United States, the relevance of masks in an epidemic setting had been well established, at least for protecting medical workers and patients. During the 1918–9 influenza pandemic, not only were masks widely used in hospitals and other medical settings, but they began to be systematically used in the community, at least in some cities.^92^ The efficacy of the masks continued to be debated and continued to be tested in the bacteriological laboratory using Flügge’s *Bacillus prodigiosus* model, showing how much this preventive technology remained tied to its origins in surgery and bacteriology in the previous century.^93^

Even in the late twentieth century, the experimental setup had changed little since the time of Mikulicz and Flügge. In a British study in the 1990s, four volunteers were placed 1m from an operating table with Petri dishes on them and asked to speak loudly and to sing in unison, with and without masks. Hundred years after the Breslau experiments, the bright red colonies of *Bacterium prodigiosus* (now under their new name *Serratia marcescens*) were still a popular indicator of the degree of impermeability of a mask.^94^

## Conclusions

The invention of the surgical mask in the late 1890s constitutes an important episode in the longer history of masking practices. The mask’s material and practical underpinnings derived mainly from the masks developed in earlier contexts. The surgical mask represents a new kind of filtering device, introduced at a point where the emerging science of bacteriology crossed paths with the novel approach to aseptic surgery. It was this confluence of surgery and bacteriology that lent new legitimacy to masking practices for anti-infectious purposes. While masks were increasingly adopted in surgery as an element of a comprehensive system against wound infection, their newly acquired legitimacy made it also easier for masks to spread to other areas. They became a research topic in bacteriological laboratories where scientists tested their efficacy for preventing various infections, notably tuberculosis. The practice, the rationale, and the method for testing their efficacy were passed on together as part of the specific technology of protection – first in hospitals in Europe and the United States, in the context of contagious diseases, then in colonial contexts and eventually in epidemics and pandemics, where they were used in the community as a means of preventing the spread of droplets containing infectious germs.

The surgical mask shaped masking practices more generally. Unlike almost all earlier masks, the new surgical mask aimed at protecting others against the emanations of the wearer, rather than protecting the wearer. One of the major elements of the germ theory was the assumption that germs could be anywhere and that even healthy people could shed pathogenic germs. The practice of preventing the transmission of germs from apparently healthy people became a key function of this type of mask. This practice would become crucial when masks came to be deployed in the community in subsequent epidemics. The understanding that healthy people – such as surgeons – could spread diseases turned the mask into a technology that could (and should, under specific circumstances) be worn by anyone.

As opposed to most of its predecessors, the surgical mask was focused exclusively on the transmission of germs, not dust and other air contaminants in general. The context of aseptic surgery, with its focus on the complete elimination of germs, gave a direction to the future development of masks. With miasmas (but also smoke, toxins, poisons), the effect on health was understood, in principle, to be dosage dependent. With germs, as understood in the late nineteenth century, it was, in principle, all-or-nothing, especially in the surgical context. Because germs were alive, they could propagate after entering the body. The mask was introduced at the moment when surgeons had switched their anti-infectious strategy from antisepsis to asepsis. This was seen as a daring move since asepsis was considered extremely precarious. Its maintenance required complete control over microorganisms. The mask was a tool to achieve this goal. In this context, the mask’s performance came to be equated with its ability to prevent the transmission of living entities that could be cultured in the laboratory using standard bacteriological techniques. From the start, the object was embedded in a set of experimental standards and practices that defined its efficacy in this sense.

The elusive goal of absolute sterility has directed the development and adoption of masks of increasingly high filtering power. In the twenty-first century, regulatory agencies have enforced standards (N95 in the US, KN95 in China and FFP2/3 in Europe) based on their filtration efficacy. The ideal of absolute sterility opened the door for growing challenges of mask efficacy, as no mask could ever reach that elusive goal, especially when worn in the community. As a result, national and international epidemic preparedness plans became increasingly critical of community masking. When the Covid-19 pandemic broke out, sanitary authorities mostly *discouraged* the use of masks by the general population, in part to protect the supply for medical workers, in part because their efficacy as a public health measure was considered unproven, before changing course in late 2020, turning the mask into the most visible sign of the global Covid-19 pandemic.

## Footnotes

1 There are a few historical accounts of masking policy and the cultural meaning of the mask, see, for example, Samuel K. Cohn Jr. , Epidemics: Hate and Compassion from the Plague of Athens to AIDS (New York: Oxford University Press, 2018), chs 19–23; Alfred W. Crosby , America’s Forgotten Pandemic: The Influenza of 1918 (Cambridge: Cambridge University Press, 2003), 101–5; Christos Lynteris , ‘Plague Masks: The Visual Emergence of Anti-Epidemic Personal Protection Equipment’, Medical Anthropology, 37, 6 (2018), 442–5730427733 10.1080/01459740.2017.1423072; Nancy Tomes , ‘“Destroyer and Teacher”: Managing the Masses During the 1918–1919 Influenza Pandemic’, Public Health Reports, 125 (2010), 48–62 20568568 10.1177/00333549101250S308PMC2862334, 56–7.

2 This is often emphasised by medical authors, see, for example, Andrea Alberto Conti , ‘Protective Face Masks through Centuries, from XVII Century Plague Doctors to Current Health Care Professionals Managing the COVID-19 Pandemic’, Acta Bio-Medica: Atenei Parmensis, 91, 4 (2020), e202012410.23750/abm.v91i4.10231PMC792751833525231; David Isaacs , ‘Mask Wearing: A Historical, Cultural and Ethical Perspective’, Journal of Paediatrics and Child Health, 57, 2 (2020), 176–732870584 10.1111/jpc.15162; Kelly Pan et al., ‘Through Plagues and Pandemics: The Evolution of Medical Face Masks’, Rhode Island Medical Journal (2013), 103, 10 (2020), 72–533261240.

3

David Edgerton , The Shock of the Old: Technology and Global History Since 1900 (Oxford: Oxford University Press, 2007), 8 . For a longer-term short survey on masks, see Bruno J. Strasser and Thomas Schlich , ‘A History of the Medical Mask and the Rise of Throwaway Culture’, The Lancet, 396, 10243 (2020), 19–20 10.1016/S0140-6736(20)31207-1PMC725530632450110.

4 We are using germ theory in the plural, following Michael Worboys , Spreading Germs: Disease Theories and Medical Practice in Britain, 1865–1900 (Cambridge: Cambridge University Press, 2000).

5 On Tyndall, Nancy Tomes , The Gospel of Germs: Men, Women, and the Microbe in American Life (Cambridge MA: Harvard University Press, 1998)11623768; John Tyndall , Fragments of Science for Unscientific People (New York: Daniel Appleton, 1871), 315 ; John Tyndall , ‘On Dust and Disease’, Proceedings of the Royal Institution, 6 (1870), 1–12 , 7–9; Worboys, *op. cit.* (note 4), 62–3, 92–3, 126, 134–8. Like many authors at the time, he used the word ‘respirator’ not in its present meaning of a device with an autonomous supply of gas, but in the sense of a filter for inhaling air.

6 Tyndall, *On Dust and Disease*, *op. cit.* (note 5), 8–9.

7

John Tyndall , ‘On Haze and Dust’, Nature, 1, 13 (1870), 339–42, 342.

8

John Tyndall , ‘On Dust and Smoke’, Notices of the Proceedings at the Meetings of the Members of the Royal Institution of Great-Britain, 6 (1871), 365–76.

9 Tyndall, *Fragments of Science for Unscientific People*, *op. cit.* (note 5); John Tyndall , ‘On Some Recent Experiments with a Fireman’s Respirator’, Proceedings of the Royal Society of London, 22 (1873), 359–61.

10

Henri Henrot , ‘Lectures’, Bulletin de l’Académie Nationale de Médecine, 43, 8 (1879), 299–300 .

11

Carl Nägeli , Die niederen Pilze in ihren Beziehungen zu den Infectionskrankheiten und der Gesundheitspflege (München: Oldenbourg, 1877), filters: 154–5, errand: 155.

12 Louis Pasteur, ‘Observations à l’occasion du procès verbal’, *Bulletin de l’Académie Nationale de Médecine*, VIII, Séance du 4 mars 1879 (1879), 171–82, 187.

13 Léon D’Ardenne, *Les microbes, les miasmes et les septicémies* (Paris: J.-B. Ballière, 1882), 42; Pasteur, *op. cit.* (note 12), 181.

14

Ferdinand Delauney , ‘Les sciences physiologiques et médicales’, Revue de France, 35 (1879), 143–62, 159.

15 Emile Decaisne, ‘Les masques contre les microbes’, *L’Univers Illustré* (1884), 522; Hector George, ‘La santé publique’, *Le Constitutionnel*, 31 March 1879, 3.

16

Edward D. Churchill , ‘The Pandemic of Wound Infection in Hospitals: Studies in the History of Wound Healing’, Journal of the History of Medicine and Allied Sciences, 20, 4 (1965), 390–404 5319602 10.1093/jhmas/xx.4.390. Many of these cases would today be diagnosed as postoperative infection. We use the word ‘wound disease’ to avoid anachronism. At the time they were often called ‘mischief’, see Michael Worboys , ‘The History of Surgical Wound Infection: Revolution or Evolution?’, in T. Schlich (ed.), The Palgrave Handbook of the History of Surgery (London: Palgrave Macmillan, 2018), 215–33, 217.

17 For this and the following passages, Thomas Schlich, ‘No Time for Statistics: Joseph Lister’s Antisepsis and Types of Knowledge in Nineteenth-Century British Surgery’, *Bulletin of the History of Medicine*, 94, 3 (2020), 394–422.

18

John Tyndall , ‘On Filtered Air’, American Journal of Pharmacy, 42 (1870), 359–62, 361.

19

Joseph Lister , ‘Address in Surgery’, British Medical Journal, 2, 556 (1871), 225–33.

20 Henrot, *op. cit.* (note 10).

21

Thomas Schlich , ‘Asepsis and Bacteriology: A Realignment of Surgery and Laboratory Science’, Medical History, 56, 3 (2012), 308–3423002302 10.1017/mdh.2012.22PMC3426977.

22 See, for example, Albert S. Landerer , ‘Die Ursachen des Misslingens der Asepsis’, Verhandlungen Der Deutschen Gesellschaft Für Chirurgie, *27. Kongress* (1898), 38–45 , 38.

23

Johann Mikulicz , ‘Ueber der neuesten Bestrebungen, die aseptische Wundbehandlung zu vervollkommnen’, Verhandlungen der deutschen Gesellschaft für Chirurgie, 27 (1898), 1–37 , 2–3.

24 Norman Davies and Roger Moorhouse, *Microcosm: Portrait of a Central European City* (2011): center: 302–4; industry: 283; university: 292; Neisser, Cohn, Koch: 297–8.

25

Hans Killian and G. Kramer , Meister der Chirurgie und die Chirurgenschulen im deutschen Raum: Deutschland, Österreich, deutsche Schweiz (Stuttgart: Georg Thieme Verlag, 1951), 187 .

26

Wojciech Kielan et al., ‘Jan Mikulicz-Radecki: One of the Creators of World Surgery’, The Keio Journal of Medicine, 54, 1 (2005), 1–7 15832074 10.2302/kjm.54.1, 4; Georg Kraft , Erinnerungen an Johannes von Mikulicz und Karl Schönborn: aus den Jugendtagen der modernen Chirurgie (Leipzig: Barth, 1926), 35.

27

Julius Neugebauer , Weltruhm deutscher Chirurgie: Johann von Mikulicz (Ulm/Donau: Haug, 1965), 128 .

28 Czerny, ‘Johannes v. Mikulicz-Radecki’, *Deutsche Medizinische Wochenschrift*, 31, 26 (1905), 1039–40.

29 Schlich, *op. cit.* (note 21).

30

J. Mikulicz , ‘Ueber Versuche, die “aseptische” Wundbehandlung zu einer wirklich keimfreien Methode zu vervollkommnen’, Deutsche Medizinische Wochenschrift, 23, 26 (1897), 409–13.

31 Mikulicz, *op. cit.* (note 23), 5.

32 Kraft, *op. cit.* (note 26), strictness: 59; janitor: 28; nobody: 38.

33

Grzegorz S. Litynski , Highlights in the History of Laparoscopy (Frankfurt/Main: Barbara Bernert Verlag, 1996), 11; W. Kozuschek et al. (eds), *Theodor Billroth*. Ein Leben für die Chirurgie (Basel: S. Karger, 1992).

34 Kraft, *op. cit.* (note 26), 54.

35 Neugebauer, *op. cit.* (note 27), 132–3.

36 Czerny, *op. cit.* (note 28).

37

Johann Mikulicz , ‘Ueber Versuche, die “aseptische” Wundbehandlung zu einer wirklich keimfreien Methode zu vervollkommnen’, Deutsche Medizinische Wochenschrift, 23, 26 (1897), 409–13, 411.

38 Czerny, *op. cit.* (note 28); Neugebauer, *op. cit.* (note 27), 168–72.

39 Mikulicz, *op. cit.* (note 23), 37.

40 Neugebauer, *op. cit.* (note 27), 24.

41 Kraft, *op. cit.* (note 26), 54.

42 Davies and Moorhouse, *op. cit.* (note 24), 298.

43 Herwarth Horn and Wolfgang Thom, *Carl Flügge (1847–1923): Integrator der Hygiene* (Wiesbaden: MHP-Verlag, 1992), 26 (note 4).

44 Czerny, *op. cit.* (note 28); Ulrich Tröhler, ‘Mikulicz-Radecki, Johannes von’, in W.F Bynum and H. Bynum (eds), *Dictionary of Medical Biography*, Vol. 4 (Westport, CT: Greenwood Press, 2007), 877–8.

45 Carl Flügge, *Grundriss der Hygiene* (Leipzig: Veit, 1908), was entirely based on Kochian bacteriology. On Flügge, see Silvia Berger , Bakterien in Krieg und Frieden: eine Geschichte der medizinischen Bakteriologie in Deutschland 1890–1933 (Göttingen: Wallstein, 2009), 52–3.

46 See the positive review of his book, Anon, ‘C. Flügge, Grundriss der Hygiene’, *Deutsche Medizinische Wochenschrift Literatur-Beilage*, 24 (1897), 167–8.

47 In 1897, Flügge published an extensive research paper on the topic. C. Flügge, ‘Ueber Luftinfection’, *Zeitschrift für Hygiene und Infektionskrankheiten*, 25, 1 (1897), 179–224.

48 See comment in Bernhard Fränkel , ‘Zur Prophylaxe der Tuberculose’, Berliner Klinische Wochenschrift, 2 (1899), 21–6.

49 Flügge, *op. cit.* (note 47), 213–4.

50
*Ibid.*, 222–4.

51 Mikulicz, *op. cit.* (note 30), 412; Mikulicz, *op. cit.* (note 23), 3; Johann Mikulicz , ‘Das Operiren in sterilisirten Zwirnhandschuhen und mit Mundbinde’, Centralblatt Für Chirurgie, 24 (1897), 713–9, 716.

52 See Schlich, *op. cit.* (note 21), which follows Bruno Latour , ‘Give me a laboratory and i will raise the world’, in K. Knorr-Cetina and M.J. Mulkay (eds), Science Observed : Perspectives on the Social Study of Science (London: Sage, 1983), 141–70.

53 Mikulicz, *op. cit.* (note 51).

54 We do not have the exact date of the first use of the face mask. Mikulicz does not mention the mask in October 1897. Mikulicz, *op. cit.* (note 37), 412; Mikulicz, *op. cit.* (note 23), 3. But he mentions the mask in a publication of July 1897, saying he has been using it for quite a while, Mikulicz, *op. cit.* (note 51), 716; Curt Schimmelbusch , Anleitung zur aseptischen Wundbehandlung (Berlin: Hirschwald, 1892), 153 . His assistant Hübener confirmed Mikulicz’spriority of the idea, Wilhelm Hübener , ‘Ueber die Möglichkeit der Wundinfection vom Munde aus und ihre Verhütung durch Operationsmasken,’ Zeitschrift für Hygiene 25 (1898), 348–72, 351.

55 Mikulicz, *op. cit.* (note 51), 716–7. Women were then not accepted as surgeons, see, for example, Claire Brock , ‘Women in surgery: Patients and practitioners’, in T. Schlich (ed.), The Palgrave Handbook of the History of Surgery (London: Palgrave Macmillan, 2018), 133–52.

56 Hübener, *op. cit.* (note 54), 351; On assistants, Schlich, *op. cit.* (note 21).

57

Johann Mikulicz , ‘Die Methoden der Schmerzbetäubung und ihre gegenseitige Abgrenzung’, Archiv Für Klinische Chirurgie, 64 (1901), 757–90.

58

W.W. Keen , ‘Transactions of the Section on General Surgery of the College of Physicians of Philadelphia’, Annals of Surgery, 27, 2 (1898), 209–27PMC142668317860548, 225–6; Thomas Schlich , ‘Negotiating Technologies in Surgery: the Controversy about Surgical Gloves in the 1890s’, Bulletin of the History of Medicine, 87, 2 (2013), 170–9723811709 10.1353/bhm.2013.0026.

59 See Curt Schimmelbusch, *Anleitung zur aseptischen Wundbehandlung* (Berlin: Hirschwald, 1892); *Ibid.*, 153–7; Schlich, *op. cit.* (note 58).

60

Alexander Fraenkel , ‘Congresseindrücke vom 27. Congress der deutschen Gesellschaft für Chirurgie in Berlin’, Wiener Klinische Wochenschrift, 21 (1898), 419–21, 420.

61 For similar scepticism in the French context, see Paul Berger , ‘De l’emploi du masque dans les opérations’, Bulletins et Mémoires de La Société de Chirurgie, 25 (1899), 187–96.

62 See, for example, Peter Poppert , ‘Ueber Seidenfadeneiterung nebst Bemerkungen zur aseptischen Wundbehandlung’, Deutsche Medizinische Wochenschrift, 23, 49 (1897), 777–80.

63 See, for example, Ludwig Rydygier , ‘Einige Bemerkungen über die auf unserer Klinik geübte Methode der Anti- und Asepsis’, Wiener Klinische Wochenschrift, 11 (1898), 993–6.

64

Anton von Eiselsberg , ‘Diskussionsbeitrag’, Verhandlungen Der Deutschen Gesellschaft Für Chirurgie, 27 (1898), 27 .

65 Painting of 1906 by Franz Skarbina (1849–1910), commissioned for Ernst von Bergmann’s twenty-fifth anniversary as chief surgeon at the Ziegelstrasse Clinic in Berlin, painting missing since 1945.

66

Berkeley Moynihan , Abdominal operations (Philadelphia: Saunders, 1906), 27 .

67 See, for example, William Watson Cheyne , A Manual of Surgical Treatment, Vol. 1 (London: Longmans Green, 1912); J. Chalmers Da Costa , Modern Surgery, General and Operative (Pennsylvania: WB Saunders, 1907), 55 ; Martin Kirschner and Alfred Schubert , Allgemeine und spezielle chirurgische Operationslehre, Vol. 1 (Berlin: Springer, 1927), 271 , 220, 241; Charles Barrett Lockwood , Aseptic Surgery (Edinburgh: Pentland, 1899), 174–5; Charles Barrett Lockwood , Aseptic Surgery (Edinburgh: Pentland, 1909), 103 . Even in the 1940s, some surgeons considered them unnecessary J. Chalmers Da Costa , The Trials and Triumphs of the Surgeon, and Other Literary Gems (Philadelphia: Dorrance, 1944), 257 .

68 Da Costa, *op. cit.* (note 67), 257.

69

Down Bros ., A Catalogue of Surgical Instruments and Appliances : Also of Aseptic Hospital Furniture Including a Large Number of Original Designs Manufactured and Sold by Down Bros., Ltd (London: Down Bros, 1906); Down Bros ., Appendix to Down Bros.’ Catalogue of Surgical Instruments and Appliances (London: Down Bros., 1910).

70

Lu Wang Adams et al., ‘Uncovering the History of Operating Room Attire through Photographs’, Anesthesiology: The Journal of the American Society of Anesthesiologists, 124, 1 (2016), 19–24 10.1097/ALN.000000000000093226569168; Matthias David et al., ‘Seit wann war die Maske Pflicht?’, Geburtshilfe und Frauenheilkunde, 81, 4 (2021), 366–9.

71

Nancy Tomes , “Destroyer and Teacher’: Managing the Masses during the 1918–1919 Influenza Pandemic’, Public Health Reports, 125 (2010), 48–62 20568568 10.1177/00333549101250S308PMC2862334, 50. On the practical character of Koch’s bacteriology, see Christoph Gradmann , Laboratory Disease : Robert Koch’s Medical Bacteriology (Baltimore: Johns Hopkins University Press, 2009).

72 Flügge, *op. cit.* (note 47), 201, 205; C. Flügge , ‘Ueber die nächsten Aufgaben zur Erforschung der Verbreitungsweise der Phthise’, Deutsche Medizinische Wochenschrift, 23, 42 (1897), 665–8, 666–7; Fränkel, *op. cit.* (note 48), 22–3; Hübener, *op. cit.* (note 54), 367–8.

73

C. Flügge , ‘Erwiderung auf Dr C. Wissemann’s Bemerkungen zu meiner Mittheilung “Ueber die nächsten Aufgaben zur Erforschung der Verbreitungsweise der Phthise”’, Deutsche Medizinische Wochenschrift, 23, 47 (1897), 758–9.

74 Fränkel, *op. cit.* (note 48), 22; Hübener, *op. cit.* (note 54), 368–71.

75 Hübener, *op. cit.* (note 54), 372.

76 Fränkel, *op. cit.* (note 48). On Fränkel, Julius Pagel , Biographisches Lexikon, hervorragender Arzte des neunzehnten jahrhunderts (Berlin: Urban & Schwarzenberg, 1901), 536–7.

77 A mask introduced by Kurt Schimmelbusch. H. Pfau, *Spezial-Preis-Liste über Instrumente für Ohr, Nase, Hals* (Berlin: H. Pfau, 1905).

78 Fränkel, *op. cit.* (note 48), 24.

79

Hermann Koeniger , ‘Untersuchungen über die Frage der Tröpfcheninfection’, Zeitschrift für Hygiene und Infektionskrankheiten, 34, 1 (1900), 119–68, Odyssey: 144; Saxonian: 148; other diseases: 162–4; gauze filters: 165; laboratory: 166.

80

Fritz Kirstein , ‘Ueber die Dauer der Lebensfähigkeit von Krankheitserregern in der Form feinster Tröpfchen und Stäubchen’, Zeitschrift für Hygiene und Infektionskrankheiten, 39, 1 (1902), 93–170 , 169.

81 See the review of his 1908 textbook *Die Verbreitungsweise und Bekämpfung der Tuberkulose*, in: *The Tubercle* 1 (5) (February 1920), 242. One example of the international spread was Norway, E. Grundt , ‘A Note on the Use of Grundt’s Mask in the Examination of Tuberculous Patients’, British Journal of Tuberculosis, 6, 4 (1912), 232–6, 235.

82

Alice Hamilton , Exploring the Dangerous Trades; The Autobiography of Alice Hamilton, M.D (Boston: Little, Brown and Company, 1943), 96–100 , on Germany, 44–51.

83

Alice Hamilton , ‘Dissemination of Streptococci through Invisible Sputum. In Relation to Scarlet Fever and Sepsis’, Journal of the American Medical Association, 44, 14 (1905), 1108–11.

84 Anon., ‘Face-Masks in Tuberculosis Work’, *The American Journal of Nursing*, 11, 12 (1911), 1045–8, 1045–6.

85

Joseph A. Capps , ‘A New Adaptation of Face Masks in Control of Contagious Diseases’, Journal of the American Medical Association, 70, 13 (1918), 910–1.

86

George H. Weaver , ‘The Value of the Face Mask and Other Measures in Prevention of Diphtheria, Meningitis, Pneumonia, etc.’, Journal of the American Medical Association, 70, 2 (1918), 76–8, 77–8.

87

Archibald L. Hoyne , ‘Fallacies of the Face Mask in the Control of the Acute Infectious Disease’, Illinois Medical Journal, 34 (1918), 136–8, 137.

88

Brewster C. Doust and Arthur Bates Lyon , ‘Face Mask in Infections of the Respiratory Tract’, Journal of the American Medical Association, 71, 15 (1918), 1216–9.

89 Charles Broquet, ‘Le masque dans la peste’, *Bulletin de La Société de Pathologie Exotique*, 4 (1911), 636–45; Lynteris, *op. cit.* (note 1); William C. Summers , The Great Manchurian Plague of 1910–1911: The Geopolitics of an Epidemic Disease (New Haven: Yale University Press, 2012); Liande Wu , A Treatise on Pneumonic Plague (Geneva: League of Nations Health Organization, 1926), 391ff.

90 R.P. Strong (ed.), ‘Studies on Pneumonic Plague and Plague Immunization’, *The Philipine Journal of Science*, VIIB, 3 (1912), 130–270, 134; Wu, *op. cit.* (note 89), 395.

91 Strong, *op. cit.* (note 90), 137–56, 187–202.

92 The most extensive study of masking policies can be found in Cohn Jr., *op. cit.* (note 1), chs 19–23; Crosby, *op. cit.* (note 1), 101–5; Tomes, *op. cit.* (note 1), 56–7.

93

W.H. Kellogg and Grace MacMillan , ‘An Experimental Study of the Efficacy of Gauze Face Masks’, American Journal of Public Health, 10, 1 (1920), 34–42 18010229 10.2105/ajph.10.1.34PMC1362677.

94

M.G. Romney , ‘Surgical Face Masks in the Operating Theatre: Re-Examining the Evidence’, Journal of Hospital Infection, 47, 4 (2001), 251–611289767 10.1053/jhin.2000.0912, 253.

